# Lutetium ^177^Lu Vipivotide Tetraxetan Efficacy and Toxicity in Advanced Prostate Cancer

**DOI:** 10.1016/j.adro.2025.101917

**Published:** 2025-10-10

**Authors:** Anthony Y. Zhang, Helaine Bertsch, Ahmed Chaudhary, Andrew Salner

**Affiliations:** a UCONN School of Medicine, Farmington, Connecticut; b Department of Radiation Oncology, Hartford HealthCare Cancer Institute at Hartford Hospital, Hartford, Connecticut

## Abstract

**Purpose:**

This retrospective study aimed to evaluate the efficacy and toxicity of lutetium ^177^Lu vipivotide tetraxetan ([LuVT], Pluvicto, Novatis Pharmaceutical Corporation), a peptide receptor radionuclide therapy, in metastatic castration-resistant prostate cancer patients treated at a single institution during the first year of Food and Drug Administration-approved clinical use.

**Methods and Materials:**

A total of 45 patients with metastatic castration-resistant prostate cancer and positive prostate-specific membrane antigen positron emission tomography imaging were treated with at least 1 cycle of LuVT therapy between September 2022 and September 2023. Clinical records were reviewed to assess prostate-specific antigen (PSA) response, imaging outcomes, and patient-reported and physician-reported toxicities. PSA responses were classified into complete, excellent (≥50% reduction), partial (10%-49% reduction), no response, and initial response with disease progression. Toxicities were graded with Common Terminology Criteria for Adverse Events v5.0 criteria.

**Results:**

Of 45 patients, 44 encompassed the final cohort (1 excluded after a single treatment before death from comorbidity). The mean age was 72.8 years and 88.9% of the cohort was White. A total of 68.9% of the cohort observed PSA biomarker improvement of ≥10%, and 55.6% with ≥50% PSA reduction. Three patients (6.7%) achieved a complete response. Imaging improvements were seen in 8 patients, including 1 with non–PSA-secreting disease. Adverse events were predominantly grade 1 and 2 severities. Most common patient-reported effects included fatigue and flare-related bone pain, with flare reactions noted in 26.7% of patients. None of the toxicities exceeded grade 2 severity. Treatment discontinuation occurred in 33.3% of patients because of a combination of progression, toxicity, lab parameters, or palliative care transition.

**Conclusions:**

LuVT therapy demonstrated consistent efficacy and tolerable toxicity in this real-world cohort, with results comparable to the VISION trial. Flare pain reactions and appetite loss emerged as prominent, although tolerable, adverse effects. Limitations include small sample size, lack of long-term follow-up, and a homogenous population with significantly advanced disease.

## Introduction

Despite advances in therapeutic modalities, metastatic castration-resistant prostate cancer remains a progressive and ultimately terminal malignancy in many patients.^1^^,^^2^ The theranostic approach has emerged as a potential treatment for metastatic castration-resistant prostate cancers (mCRPCs), offering precise diagnosis and staging, and targeted therapy for patients with “high likelihood of recurrence and metastasis.”^3^ This is because of high expression of prostate-specific membrane antigen (PSMA) in mCRPCs.^4^ High PSMA levels have been found to be a prognostic factor for decreased overall survival of mCRPC patients.^5^ PSMA is targeted via radioligand lutetium ^177^Lu vipivotide tetraxetan ([LuVT], Pluvicto, Novartis Pharmaceutical Corporation) to deliver beta-particle radiation to the PSMA-expressing tumor cells.^1^ Originally studied in the VISION trial, there was a significant improvement in overall survival by 4 months, and markedly delaying symptoms from bone metastasis common in these patients. The Vision trial is a pivotal phase 3 study evaluating the efficacy of 177 Lu-PSMA-617, a radioligand therapy, in patients with metastatic castration-resistant prostate cancer.^1^

The field of radiopharmaceuticals is characterized by an integrated diagnostic and therapeutic paradigm. The former is characterized by molecular imaging of the cancer with a diagnostic positron emission tomography (PET) imaging agent such as ^68^Ga bound to the molecule targeting the cancer cells. The latter is a therapeutic radioligand such as ^177^Lu bound to the same molecule.^6^ Because of elevated PSMA expression in mCRPR, tumor cells can be effectively targeted via LuVT. Given the heterogeneous nature of prostate cancer, PSMA PET scan serves as a critical predictive tool for determining response to LuVT therapy. Prostate tumors vary in PSMA expression and physical tumor texture between patients.^7^ The benefit of pretreatment PSMA PET scan is 3-fold, because it identifies eligible patients presenting with PSMA positive disease, delineates the location and extent of disease, as well as serving as a baseline measure for posttreatment response.^8^

As a result of the VISION trial findings, LuVT was approved by the United States Food and Drug Administration as a treatment for patients with mCRPC in 2022.^4^ Prior to the integration of theranostic strategies in mCRPC management, treatment options were notably limited. Predominantly, mCRPC patients were treated with androgen deprivation therapy, androgen receptor pathway inhibitors such as enzalutamide and abiraterone, taxane chemotherapy, and immunotherapy.^4^ The addition of LuVT therapy provides another promising treatment for mCRPC patients. In the TheraP study comparing LuVT therapy to taxane chemotherapy, it was found that LuVT therapy led to a significantly greater PSA response proportion than taxane chemotherapy.^6^ The VISION trial elucidated the efficacy of LuVT therapy via PSA response and imaging improvement measures. Further studies revealed additional potential biomarkers to track the efficacy of LuVT therapy. An international multi-institutional study noted PSA decrease following 2 therapy treatments and alkaline phosphatase levels < 220 U/l were indicative of longer overall survival.^4^

The use of LuVT therapy has shown efficacy in prior studies but comes with certain toxicity risks. Prior LuVT therapy trial noted an increase of toxicity effects following increased exposure, with increased fatigue, increased dry mouth symptoms, and myelotoxicity.^1^ In addition to an increase in quantity of adverse effects, the severity of adverse effects also increased. The VISION trial noted a higher incidence of grade 3 adverse events in using LuVT therapy than without.^1^ However, this increased severity in adverse effects did not significantly affect patient quality of life. A small risk of secondary malignancy is certainly one of the longer-term concerns.

Although LuVT therapy seems effective with minimal adverse effects, there is more investigation to be done. This retrospective study investigated the efficacy of LuVT therapy via both imaging results and PSA response, as well as exploring the toxicity of this therapy in patient-reported and physician-reported categories, in the authors’ institution’s first treatment year. Because our cohort spans the first year of Novartis production following Food and Drug Administration approval of agent, intermittent manufacturing and transport logistics from Italy occasionally required rescheduling of individual treatment days, despite standard handling, storage, and radiation-safety protocol.

## Methods and Materials

This retrospective study was approved by the institutional review board at our institution, and all patient data were deidentified in accordance with Health Insurance Portability and Accountability Act regulations. This study focused on 45 patients treated with LuVT from September 2022 to September 2023. Data collection for each patient included prostate cancer history, LuVT therapy data, lab test results, imaging results, and reported therapy adverse events. Prostate cancer history categories included cancer stage and grade, date of disease progression, and prior therapies. LuVT therapy data included therapy timeline and PSA test results coinciding with each therapy date. Lab tests collected include serial complete blood counts, renal function assessments (creatinine), and hepatic function panels. Imaging results included bone scans, computed tomography and magnetic resonance imaging scans, and PSMA PET imaging results. Lastly, adverse events were recorded as patient reported or as physician reported via abnormal lab test values according to Common Terminology Criteria for Adverse Events v5.0 standards.

This data was evaluated to assess LuVT efficacy and actual adverse events. Efficacy was evaluated primarily by PSA response to treatment, with imaging response assessed primarily for the single patient who remained PSA nonsecreting. The PSA biomarker was recorded following each therapy date and analyzed for change between the pretreatment PSA to the last recorded treatment PSA. PSA response was categorized into 5 categories: complete response (PSA became undetectable), excellent response (PSA decrease > 50%), partial response (PSA decrease of 10%-50%), no response, and initial response with treatment disease progression. To determine a treatment as effective, a PSA reduction threshold of at least 10% was established as clinically meaningful. Patients with initial response > 10% followed by disease progression were classified in an individual category. Time to PSA50 was defined as the first treatment visit at which PSA had fallen ≥50% from the pretreatment baseline and was summarized both by treatment cycle number (1-6), and as calendar days from pretreatment date to outcome date. The PSA-nonsecreting patient was excluded from PSA-based endpoints. Therapy efficacy was further evaluated qualitatively via comparative imaging response with 3 categories: disease improvement on imaging, positive PSA response correlated to imaging, and negative PSA response correlated to imaging. Imaging was used to assess response because of sample containing one patient with non–PSA-secreting tumor.

Toxicity of LuVT therapy was analyzed via patient reported adverse events and via physician reported adverse events. Patient reported adverse events were graded via the Common Terminology Criteria for Adverse Events (CTCAE) v5.0 guidelines into grades of mild, moderate, and severe to evaluate the severity of toxicity among the patient sample. Patient reported adverse events were carefully analyzed to remove effects prior to therapy. Physician reported adverse events were collected via lab values and included low Complete Blood Count value, abnormal liver function tests, low hematocrit, fatigue, and worsening performance status. Physician reported adverse events were scored using CTCAE guidelines and were graded into transient and improving over time categories.

## Results

The retrospective study analyzed 45 patients who received at least 2 LuVT therapy treatments. One patient was omitted from the analysis because of only receiving 1 treatment prior to death by comorbidity. The racial makeup of study participants included 40 Caucasians, 3 African Americans, and 2 “Other.” Ethnicity of the patient population consisted of 1 Hispanic and 44 non-Hispanic. Age range was 49 to 85 with a mean age of 72.8 (see Table 1). All 45 patients had mCRPCs, stage IV disease with positive tumor avidity findings on PSMA PET imaging, and were treated with LuVT therapy. Thirty-four (75.6%) patients had received definitive treatment with prostatectomy or radiation therapy, and 42 (93.3%) patients had prior systemic therapy with androgen deprivation therapy, androgen receptor pathway inhibitors, and taxane chemotherapy. All 45 patients received definitive treatment or prior systemic therapy prior to LuVT therapy. Cohort baseline PSA median was 51.6 ng/mL (IQR, 14.06-160.20). Although the majority of Eastern Cooperative Oncology Group performance status was 0-1, several patients had more advanced Eastern Cooperative Oncology Group status of 2 within this cohort.

**Table 1:** Patient characteristics

Characteristic	Value
Age, y – mean (range)	72.8 (49-85)
Race – White – n (%)	40 (88.9%)
Race – African American – n (%)	3 (6.7%)
Race – Other – n (%)	2 (4.4%)
Ethnicity – Hispanic/Latino – n (%)	1 (2.2%)
Ethnicity – not Hispanic/Latino – n (%)	44 (97.8%)
**Prior therapy – n (%)**	
Definitive local therapy (Prostatectomy/Radiation therapy)	34 (75.6%)
Systemic therapy (ADT/ARPIs/Chemotherapy)	42 (93.3%)
**Baseline lab features**	
PSA, ng/mL – median (IQR)	51.6 (14.06-160.20)
Hematocrit, % - median (IQR)	34.1% (30.6%-36.75%)
Creatinine, mg/dL – median (IQR)	0.84 (0.73-1.1)
Time from initial diagnosis to LuVT Start, months – median (IQR)	81 (54-102)
Time from first metastasis to LuVT Start, months – median (IQR)	51 (35.5-80.8)

*Abbreviations:* ADT = androgen deprivation therapy; ARPI = androgen receptor pathway inhibitor; LuVT = lutetium Lu 177 vipivotide tetraxetan; PSA = prostate-specific antigen.

Treatment compliance showed that 66.67% completed therapy, 17.78% died during treatment, and 15.55% had therapy withheld because of complications. Patients stopped treatment prematurely for several factors. 8 patients passed away during treatment time, 2 patients stopped because of higher grade adverse effects, 3 patients because of Complete Blood Count levels below permissible levels, and 2 patients because of opting for palliative care. Of the 8 deceased patients, 7 died from progressive disease and 1 died from myocardial infarction which did not appear linked to therapy.

Efficacy of LuVT treatment was primarily assessed by comparing PSA biomarker levels before and after treatment to evaluate response. PSA response across treatment was categorized into 5 categories. Three patients (6.7%) had a complete response, 22 patients had an excellent response (48.9%), 3 patients had a partial response (6.7%), 16 patients with no response (37.8%), and 3 patients with initial response but progression during treatment. Three of the patients with initial response subsequently progressed during treatment. The overall response rate was 68.9%. Out of 45 patients, 1 patient was omitted from the PSA response as the patient was PSA undetectable prior to and throughout the entire treatment.

**Figure 1:**
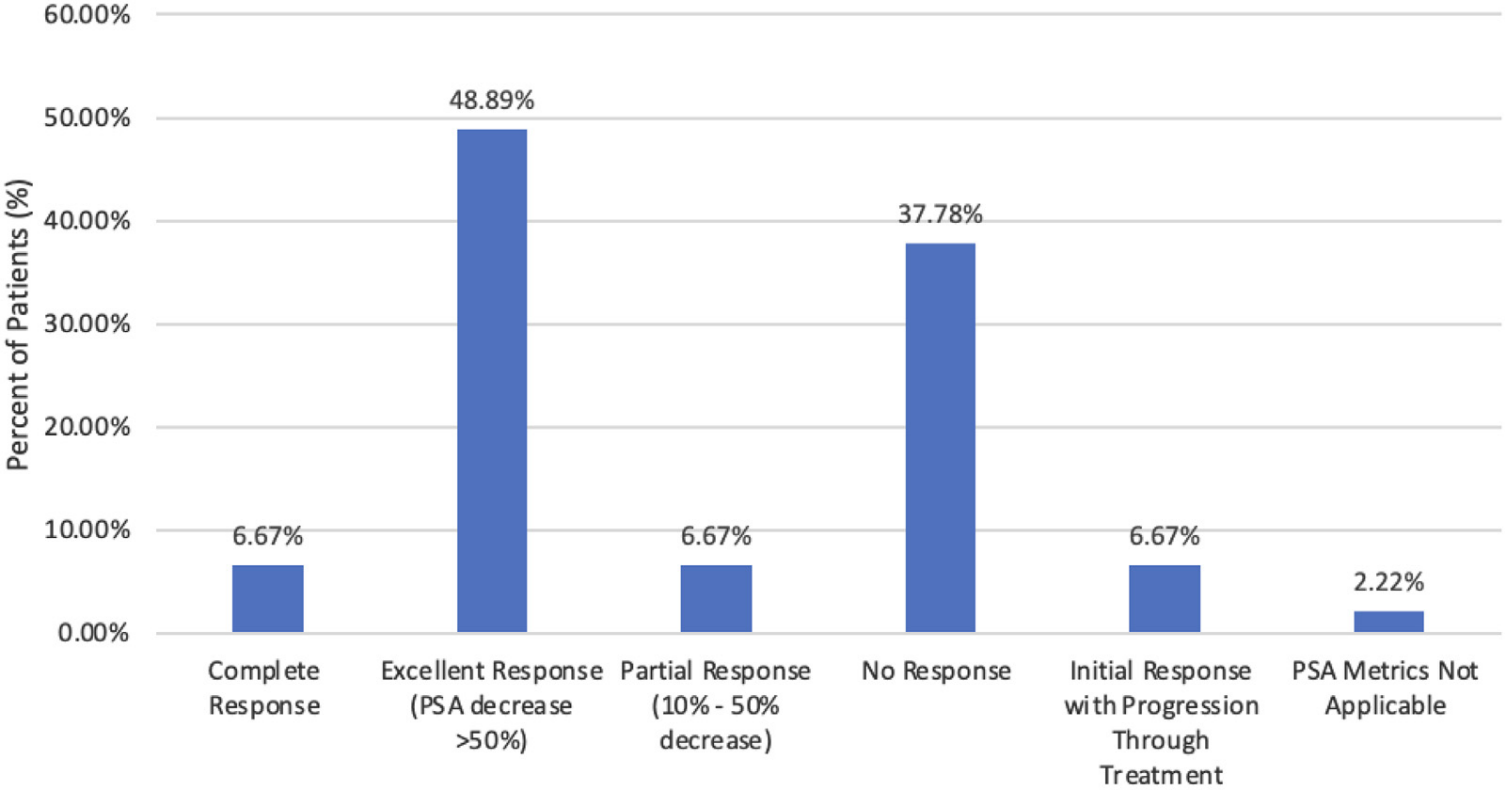
PSA responses to LuVT treatment. *Abbreviation:* PSA = prostate-specific antigen.

PSA response over time was assessed across treatment cycles. As seen in Fig. 1, among 44 patients, 23 achieved a PSA decline of 50% or greater (PSA50). PSA50 occurred after 1 treatment cycle in 5 patients (26.3%), 2 cycles in 12 patients (63.2%), 3 cycles in 2 patients (5.3%), 5 cycles in 1 patient (5.3%), and 6 cycles in 3 patients. The median number of treatments to PSA50 was 2 cycles (IQR, 2-2.5). Among responders, the median time from treatment initiation to PSA50 was 78.5 days (IQR, 65-116 days). In patients with excellent PSA response, PSA50 was achieved in a median of 2 treatment cycles (IQR, 0.5-3), with a median of 81 days (IQR, 68.5-145.5 days). In 2 complete response patients, they achieved PSA50 after treatment cycles 1 and 2. Age did not differ significantly between responders and nonresponders (mean 74 vs 72.4 years, *P* = .49), nor was there significant difference across PSA response categories no PSA response, partial response, excellent response, and complete response (analysis of variance *P* = .9; Kruskal–Wallis *P* = .94).

Efficacy of LuVT treatment for a subsection of the sample was further assessed for patients via imaging. Not all 45 patients were assessed with imaging because of uncovered costs of imaging posttreatment. Imaging disease progression was tracked via bone scan and PSMA PET scan comparisons. Eight patients had disease improvement following LuVT treatment, with 2 of those patients having had no PSA response to treatment. The patient with undetectable PSA markers at baseline demonstrated PSMA PET imaging radiographic improvement across all 6 treatment cycles, with notable reduction in lymphadenopathy and bone sclerosis.

**Figure 2:**
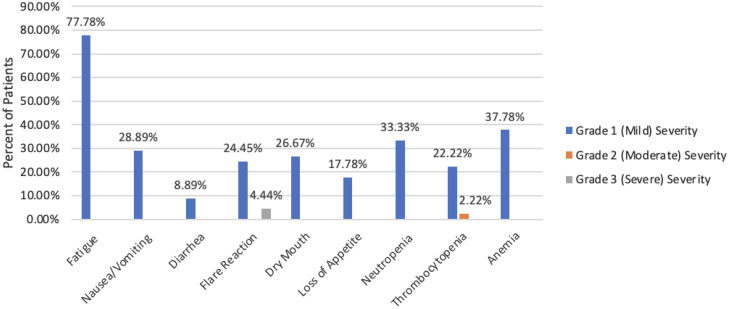
LuVT adverse events (CTCAE v 5.0). *Abbreviation:* LuVT = lutetium Lu 177 vipivotide tetraxetan.

Fatigue emerged as the most frequently reported and impactful adverse event, with over 50% of patients reporting mild symptoms and 16% reporting moderate-grade fatigue following treatment (see Fig. 2). Flare reaction-related pain was noted in a significant number of patients. Three patients had higher grade patient-reported flare-related pain, 2 of whom required treatment delays. A total of 26.7% of patients had flare reactions characterized by pain increase following early treatments (usually first and second). Increased pain was noted to have variability across grade severity and led to 1 noncompliant patient (see Fig. 2).

**Figure 3:**
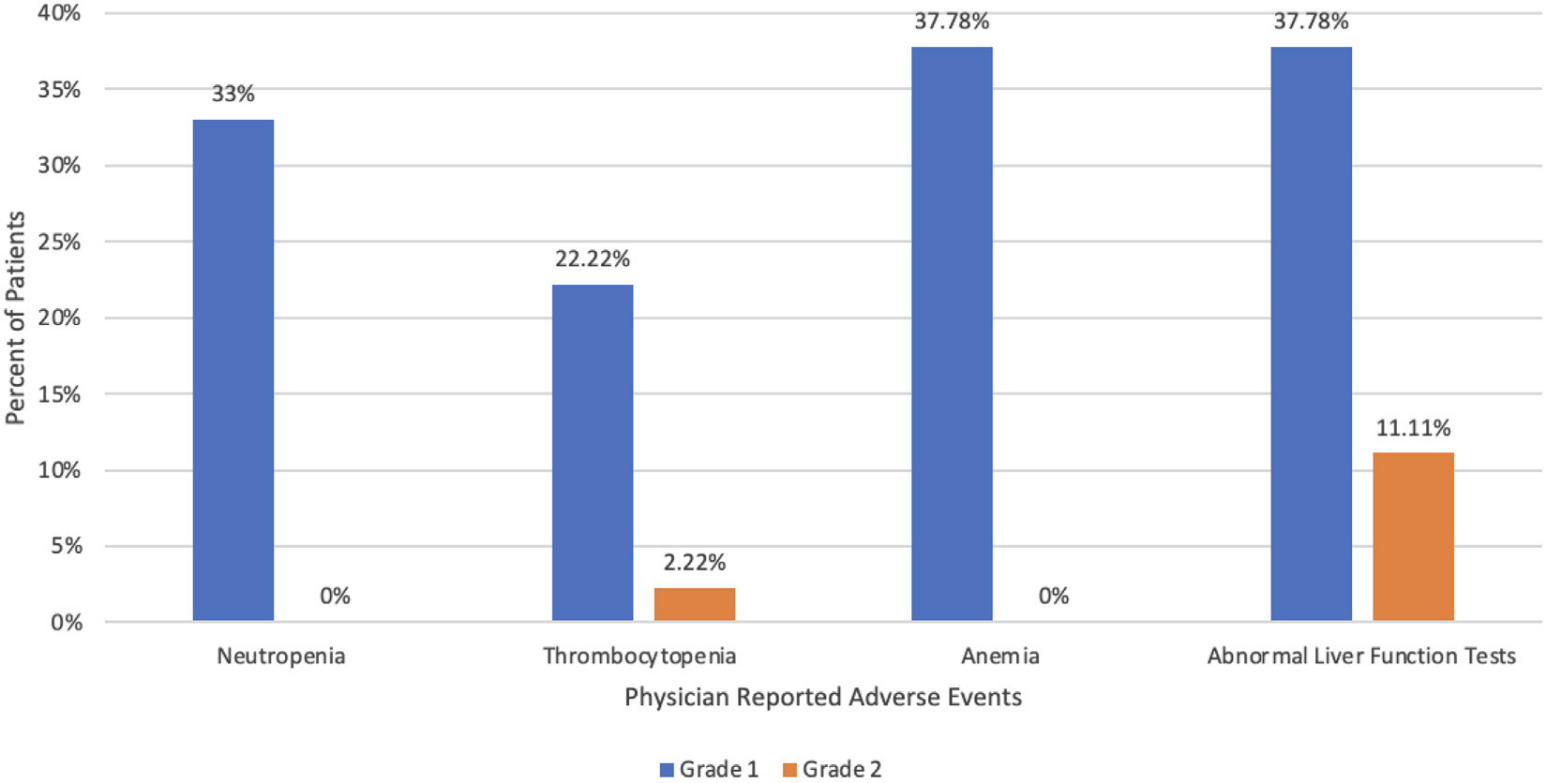
Lab value-based toxicity (CTCAE v 5.0).

Physician-reported adverse events were sorted by most common categories of neutropenia, thrombocytopenia, anemia, and abnormal liver function tests. Across the physician-reported adverse events no patients experienced toxicities above grade 2. However, 3 patients with physician-reported adverse events had treatment withheld because of below permissible lab levels. Physician-reported adverse events were common and low grade from LuVT therapy (see Fig. 3). Abnormal liver function was noted with highest severity, with 11.11% of patients having grade 2 abnormal liver function.

## Discussion

Prior to the initiation of theranostic therapy, advanced castration-resistant prostate cancer patients had very limited treatment options to reduce cancer progression and symptoms. Forty-five patients with advanced castration-resistant prostate cancer were treated with LuVT therapy at our institution. One patient was omitted because of receiving only 1 treatment prior to death because of comorbidity. One patient was noted to be PSA negative but was included and analyzed via imaging comparisons.

LuVT compliance was measured by patient completion of treatment and stopped treatment categories. Of note, 33.3% of patients stopped treatment for numerous reasons. In addition, another 2 patients opted to stop treatment with progressive advanced disease for palliative care focusing on better quality of life. Because this was the first group of patients treated with a newly approved therapy, the group included more patients with substantially advanced disease exhausted by all other treatment options. Eleven percent of patients had treatment withheld because of toxicities, 3 because of severe patient-reported toxicity and 2 because of lab values falling below predetermined parameters to prevent potential severe effects. Of 8 deceased patients during treatment, 0 deaths were treatment-related, 7 deaths were disease-related, and 1 death because of myocardial infarction.

**Figure 4:**
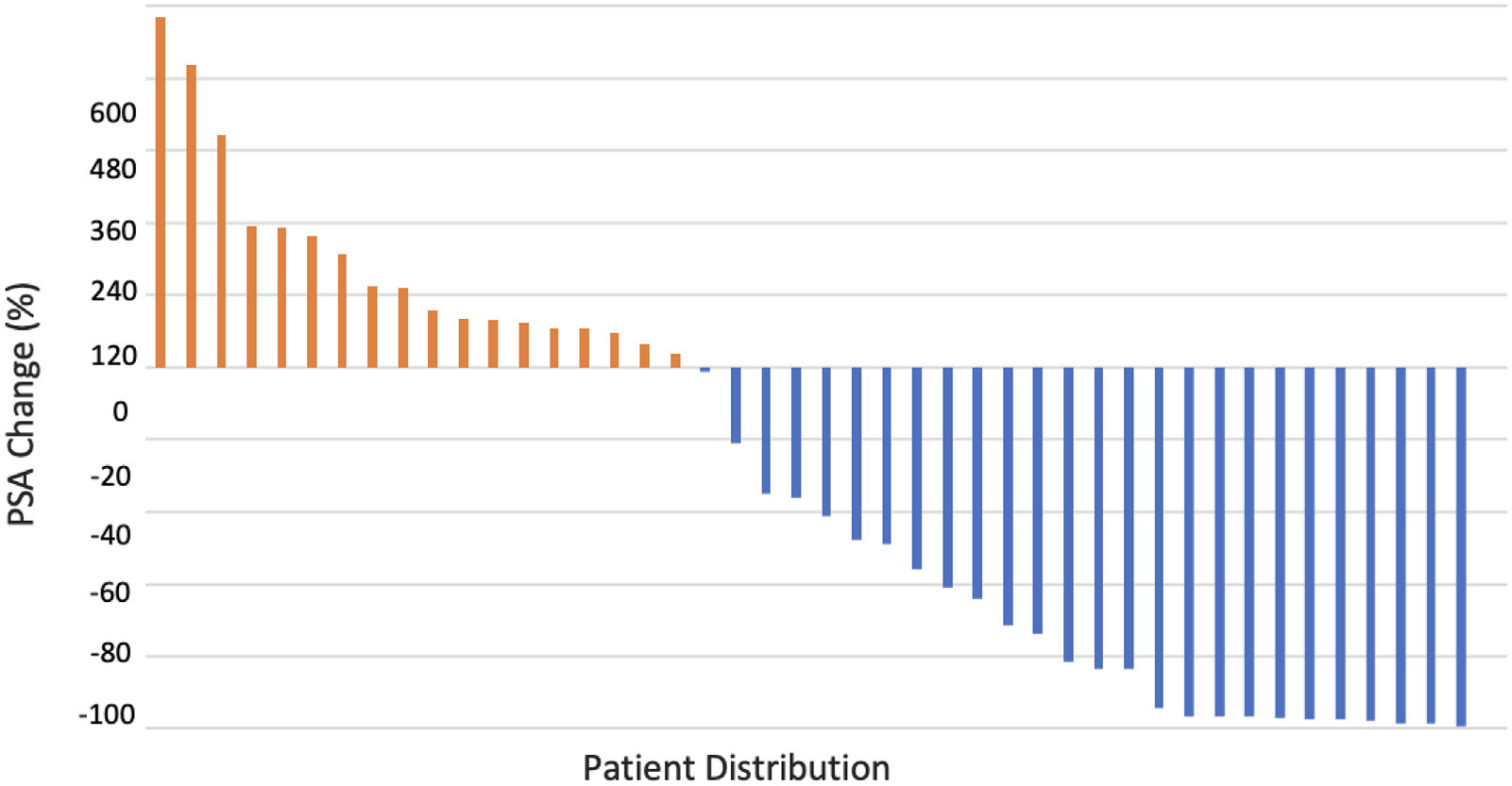
PSA change over treatment course. *Abbreviation:* PSA = prostate-specific antigen.

LuVT efficacy was demonstrated with 68.9% of patients with quantitative response to therapy and 62.2% of patients having positive response through treatment end. Through these positive PSA results, LuVT therapy was able to reduce PSA-marked disease progression in 62.2% of patients. Remarkably, 55.6% of patients had PSA response of 50% or greater, indicating excellent or complete responses with LuVT therapy (see Fig. 4). When compared to VISION trial, our study had complete response in 3 patients (6.7% vs 9.2%, n = 17), and 62.2% positive partial response when compared to VISION trial 41.8%^2^. However, these cross-study comparisons are descriptive and should be interpreted cautiously provided our small, single-center cohort, and is therefore hypothesis-generating rather than definitive.

In exploratory analysis, age was not associated with PSA response, with mean and median ages similar between responders and nonresponders, across partial, excellent, and complete response categories. This suggests that efficacy of LuVT therapy did not appear to be driven by patient age, although the study may be underpowered to detect subtle subgroup differences.

Although the initial study design included analysis of posttreatment imaging to complement PSA response metrics, access to authorized follow-up PET imaging was frequently restricted because of insurance coverage limitations. Many patients faced insurance coverage denial for posttreatment PET scans, resulting in inconsistent imaging follow-up. Therefore, imaging analysis is omitted from the primary analysis, except for the singular case where imaging was used exclusively to evaluate response in a patient with a non–PSA-secreting tumor.

The most common patient-reported adverse events included fatigue, nausea, dry mouth, and flare reaction pain, which were primarily mild (grades 1 and 2) in CTCAE v5.0 severity. Physician-reported adverse events commonly involved neutropenia, thrombocytopenia, anemia, and abnormal liver function tests mild in severity throughout treatment. Long-term toxicity has not been evaluated because of the lack of supporting data thus far. When compared to VISION trial, our results demonstrated fewer grade 3 or higher adverse effects, (6.67% vs 52.7%), with 93.3% of patient-reported adverse events grade 2 or lower, albeit within a small, retrospective cohort.^2^

Variance in therapy responses can be attributed to tumor heterogeneity, including differences in PSMA expression in mCRPC, which influence both the extent of therapy response and the likelihood of early progression.^9^ Previous studies have shown varied response to LuVT, such as poor treatment response because of low PSMA uptake.^7^ Because of a combination of heterogeneity between patients and molecular attributes of each tumor, variation in treatment response is expected.^7^

A noteworthy finding of the study was the relatively high incidence of flare reactions following initial LuVT treatments. Flare reactions, defined by a transient exacerbation of symptoms, usually osseous pain, were reported in 26.7% of patients. Although these reactions were mild to moderate in severity, flare reactions occasionally contributed to treatment delays and patient noncompliance. This phenomenon is consistent with prior observations in lutetium 177Lu dotatate therapy, where flare reactions were similarly documented and postulated to arise from radiation therapy-induced peritumoral inflammation.^3^ Recognizing flare reactions as a potential early treatment effect may help clinicians better anticipate and manage symptoms during therapy initiation, potentially improving treatment adherence. Short courses of steroids can be effectively used in appropriate situations to treat or prevent these flare reactions.

## Conclusions

In this retrospective analysis, the overall efficacy and toxicity findings were consistent with data found from larger studies such as the VISION trial. Complete response rates were comparable to the VISION trial, with 6.7% in authors’ study versus 9.2% in VISION.^1^ Adverse effects were within an acceptable range and yielded similar results to the VISION trial. In addition to the VISION trial’s documentation of fatigue, xerostomia, and nausea as primary adverse effects, this study further highlights flare reaction pain and appetite loss as notable side effects of LuVT therapy.^1^ Severity of adverse events were similar, with predominant grade 1 or 2 toxicities, and no toxicities beyond grade 3 in the retrospective study.

Limitations of this study included a homogenous cohort with significantly advanced disease and small sample size. Among the patient population, 88.89% were White, making it difficult to generalize this data to the advanced castrate-resistant prostatic cancer population. Furthermore, the sample included patients with very advanced prostatic cancer, notably because this treatment was new and offered some hope for those whose disease had become resistant and progressive. Therefore, we observed some patients who discontinued treatment either because of death from comorbidities or in seeking palliative care. In retrospect, patients with very advanced disease may not be the ideal study sample because of poor performance status (see Fig. 4).

## Disclosures

Andrew Salner reports a relationship with Lantheus Holdings Inc that includes: consulting or advisory. The other authors declare that they have no known competing financial interests or personal relationships that could have appeared to influence the work reported in this paper.
